# Different femoral tunnel placement in posterior cruciate ligament reconstruction: a finite element analysis

**DOI:** 10.1186/s12891-023-06161-y

**Published:** 2023-02-04

**Authors:** Bing Wang, Yongjie Ye, Long Yao, Ancheng Wei, Xin Huang, Zhiqiang Wang, Xiaojun Yu

**Affiliations:** 1 Orthopedic, Suining Central Hospital, No.127, Desheng West Road, Suining, 629000 People’s Republic of China; 2 North Sichuan Medical College, No. 234, Fujiang Road, Nanchong, 637100 People’s Republic of China; 3 Orthopedic, Chengdu Fifth People’s Hospital, NO.33, Mashi Street, Chengdu, 611130 People’s Republic of China; 4 Chengdu University of Traditional Chinese Medicine, No. 1166, Liutai Avenue, Chengdu, 610075 People’s Republic of China

**Keywords:** Critical corner, Posterior cruciate ligament, Reconstruction, Finite element, Biomechanics, Knee joint injury

## Abstract

**Background:**

At present, there is no consensus on the optimal biomechanical method for Posterior cruciate ligament (PCL) reconstruction, and the “critical corner” that is produced by the femoral tunnel is currently considered to be one of the main reasons for PCL failure. Thus, the purpose of this study was to identify one or several different tunnels of the femur, thereby reducing the influence of the "critical corner" without reducing the posterior stability of the knee.

**Methods:**

CT and MRI data of the knee joint of a healthy adult man were collected, and computer-related software was used to reconstruct the finite element model of the knee joint, to provide different properties to different materials and to allow for the performance of a finite element analysis of the reconstructed model. The position of the femoral tunnel was positioned and partitioned according to anatomical posture, and three areas were divided (the antero-proximal region, the antero-distal region and the posterior region). In addition, we applied a posterior tibial load of 134 N to the reconstructed model, recorded and compared different tunnels of the femur, conducted peak stress at the flexion of the knee joint of 0°, 30°, 60° and 90°, and elicited the displacement of the proximal tibia.

**Results:**

Among the 20 different femoral tunnels, the graft peak stress was lower in tunnels 4, 12 and 18 than in the PCL anatomical footpath tunnel 13, especially at high flexion angles (60° and 90°). These three tunnels did not increase the posterior displacement of the proximal tibia compared with the anatomical footpath tunnel 13.

**Conclusion:**

In summary, among the options for PCL reconstruction of the femoral tunnel, the tunnels located 5 mm distal to the footprint and 5 mm anterior to the footprint could reduce the peak stress of the graft; additionally, it may reduce the "critical corner" and was shown to not reduce the posterior stability of the knee joint.

## Background

Posterior cruciate ligament (PCL) injury is a common clinical problem. PCL tears comprise 3% of outpatient knee injuries and 38% of acute traumatic knee hemarthroses [1]. Some scholars believe that isolated injuries account for about 40% of PCL injuries [2]. Patients with isolated grade I or II PCL injury, or grade III injury with mild symptoms or low activity needs are treated nonoperatively and typically have good functional outcomes (including knee stability, quadriceps values measurement, etc.) [3–6]. However, for symptomatic complete and combined PCL injuries, surgery is recommended to restore joint stability and improve function. Because, persistent instability of the knee due to posterior cruciate ligament tears, knee pain, limited mobility and osteoarthritis will occur in the future [7–10]. PCL reconstruction has a high failure rate due to incorrect tunnel placement, selection of inappropriate surgical techniques, grafts, timing of postoperative rehabilitation, and the presence of other associated complications (infection, pain, etc.) [11–16]. The results of a previous study showed that only 50–82% of the patients who underwent PCL reconstruction were able to return to the preinjury activity level [17]. In addition, the incidence of joint degeneration after PCL reconstruction has been reported to be between 15 and 60% [18].

At present, there is no consensus on the reasons for the different biomechanical effects of PCL reconstruction, and the probable cause of PCL reconstruction failure is theorized to be incorrect tunnel location [11, 12, 19]. The acute flexion angle at the intra-articular interface between the graft and the tibial tunnel is known as the "killer turn" [20, 21]. Analogous to the ‘‘killer turn’’, an acute graft bending angle at the intra-articular femoral tunnel may result in early graft failure; this scenario is known as the “critical corner” or “acute angle”. The “critical corner” or “acute angle” from the femoral tunnel can cause graft failure [22, 23]. At present, several studies have shown that different placements of the femoral tunnel can lead to different biomechanical results [24–29]. However, until now, we have not identified one or more ideal tunnels, as well as their specific coordinates, that can effectively reduce the “critical corner” or “acute angle”.

Therefore, the present study aimed to confirm one or more ideal femoral tunnel points during PCL reconstruction that can reduce the “critical corner” via the three-dimensional (3D) finite element (FE) analysis. We hypothesized that these new points of the femur are useful for the correct-placing of the femoral tunnel; additionally, in these new points, the graft peak stress can be reduced.

## Materials and methods

### Establishment of a three-dimensional finite element model of the knee joint

A 35-year-old adult healthy male volunteer (175 cm, 75 kg) participated in the study and signed an informed consent form. The volunteer had no history of knee infection, injury or other conditions that may have affected the experiment. The subject was placed in the supine position, and the knee joint was fixed with a knee brace to reduce the movement of the knee joint during data collection.

Patient knee data were collected by using Siemens ingenuity core 64-slice spiral computed tomography (CT) and 1.5-T dual gradient nuclear magnetic resonance imaging (MRI, Siemens MAGNETOM Aera). CT scanning parameters(120kv, automatic milliampere-second scanning, scanning layer thickness 5 mm, reconstruction layer thickness 1 mm, matrix 512 × 512, radiation dose 20mgy (CTDIvol)); MRI scanning parameters (t1 sagittal bit, TSE sequence, layer thickness 3.5 mm, layer spacing 0.35 mm, TE: 12 ms, TR: 690 ms; PD sagittal lipid press, TSE sequence, layer thickness 3.5 mm, layer spacing 0.35 mm, TE: 35 ms, TR: 2980 ms; pd coronal lipogram, TSE sequence, layer thickness 3.5 mm, layer spacing 0.35, TE: 35 ms, TR: 2500 ms; t2-axis lipid press, TSE sequence, layer thickness 4 mm, layer spacing 0.4 mm, TE: 85 ms, TR: 3400 ms).

The volunteer's CT and MRI examinations showed no knee skeletal developmental deformities, meniscus injuries, periknee ligament injuries, and articular cartilage injuries. Knee CT data (distal femur, proximal tibia, proximal fibula and patella) and MRI data (cartilage, meniscus, anterior cruciate ligament, posterior cruciate ligament and medial and lateral collateral ligaments) were imported into Mimics Research 21.0 and 3D Slicer 4.10.2, respectively. The exported data were saved in the STL format.

The obtained STL data were imported into ZBrush 2019 to beautify and adjust the corresponding model. The data were output in OBJ format and imported into Rhino 7 for grid representation design. 3D models of different knee joints were made according to the experimental requirements.

**Fig. 1:**
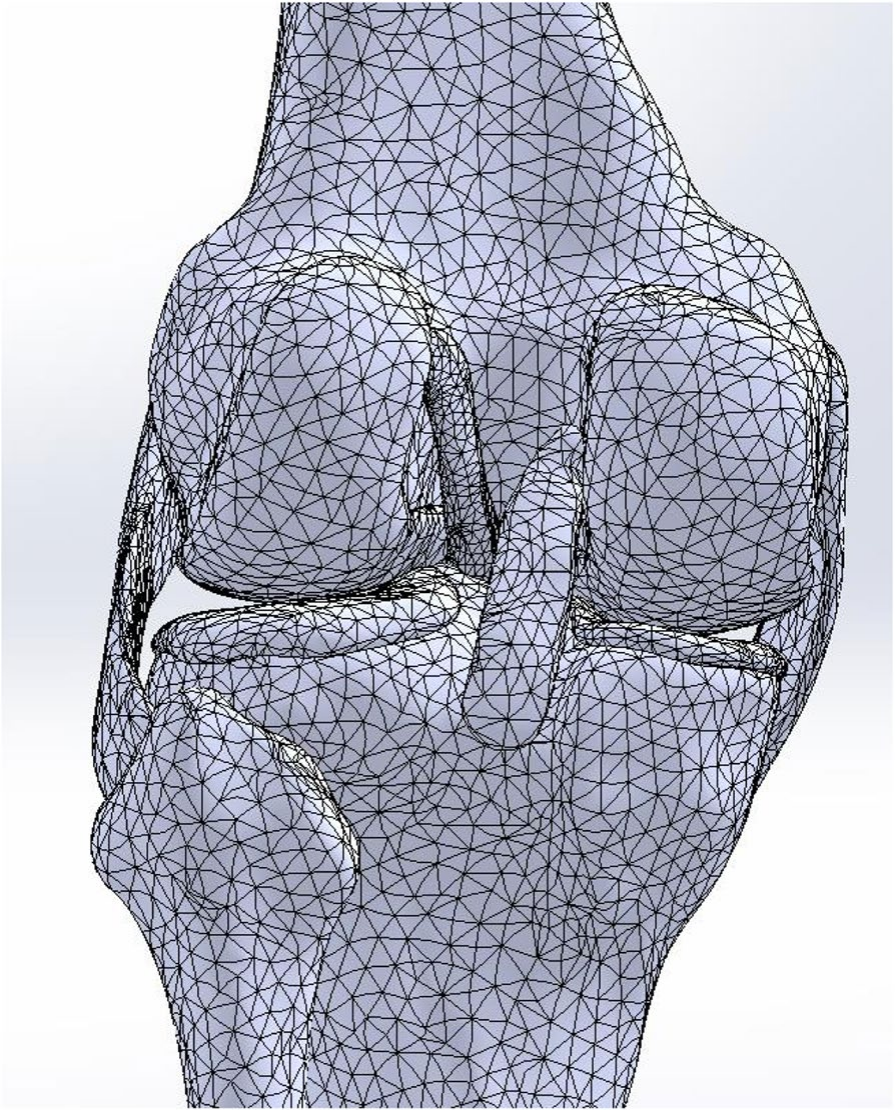
Geomagic Design X 64 was used to mesh the three-dimensional geometric model of the knee joint. The model was divided into 2,599,237 cells and 3,727,928 nodes

Via Geomagic Design X 64, the mesh was divided and checked to make the mesh smoother and more flexible, thus ensuring a high-quality surface to complete the experiment. The model was divided into 2,599,237 cells and 3,727,928 nodes (Fig. 1). The finite element analysis software SOLIDWORKS 2018 was used to analyze the 3D geometric model of the knee joint.

### Material modeling

The femur and tibia are considered to be rigid because their stiffness is significantly higher than that of soft tissue [30]. According to previous literature reports, articular cartilage and meniscus are considered to be a single-phase linear elastic and isotropic material with elastic moduli of 5 MPa and 59 MPa, respectively, and the Poisson's ratios of articular cartilage and meniscus are 0.46 and 0.49, respectively [31, 32]. We set the ligament as a homogeneous, continuous, hyperelastic, rubber-like material, which represents the nonlinear stress‒strain relationships [33]. Finally, to simulate the real-life knee structure, each accessory and tissue were anatomically linked.

### Establishment of models with different bend angles

**Fig. 2:**
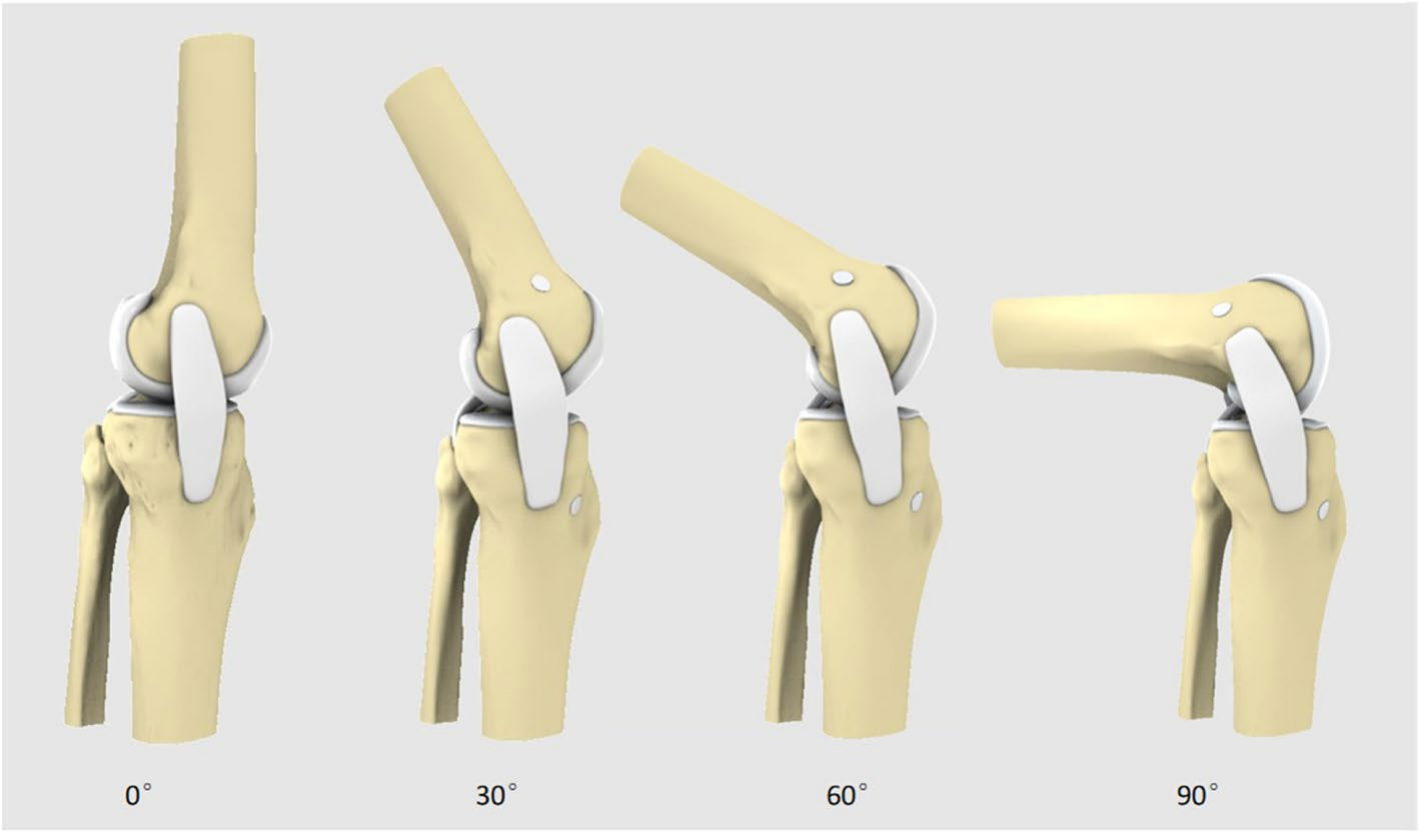
The computer reconstructed model of the knee joint is a lateral position plate of 0°, 30°, 60° and 90° of the knee

Flexion in the reconstructed knees was simulated as follows. After the PCL reconstruction model was created, the femur was immobilized, and the tibia was rotated posteriorly by using computer software. Different knee joint models with knee bends of 30°, 60° and 90° were obtained (Fig. 2).

### Tibial and femoral tunnel placements

**Fig. 3:**
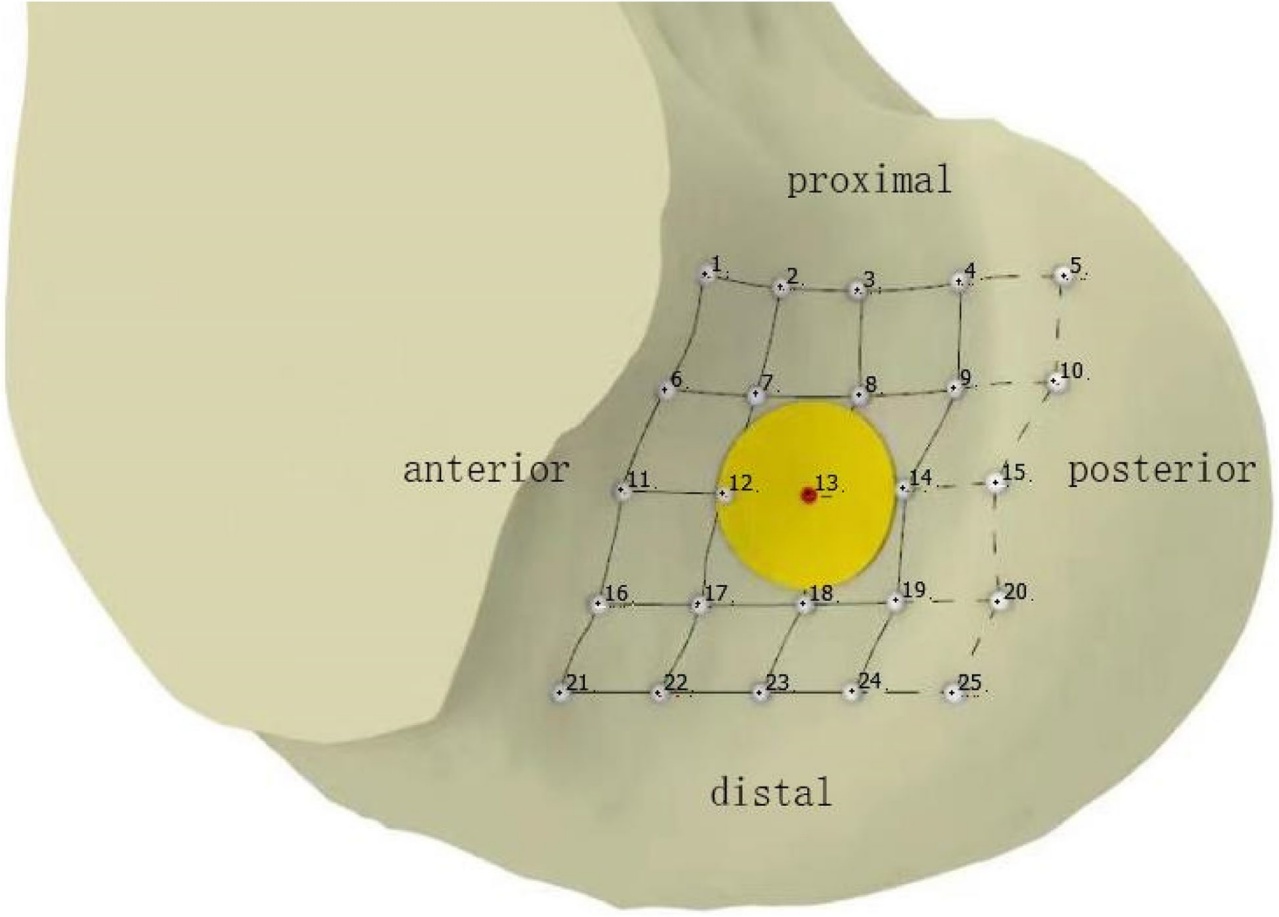
Anatomical posture was used for positioning, and the tunnels were digitally arranged. Tunnel 13 is located in the middle of the footprint and is marked in red. Yellow is the circular tunnel with a diameter of 9 mm

The tibial tunnel is located at the tibial footprint of the PCL. Based on our team's previous experiments, we adopted a similar tunnel design for the femur [34]. In the distal femur, with the PCL anatomical site as the center, the distance of 5 mm was in the distal–proximal direction and in the direction. At the intersection of each point and the bone surface, femoral tunnels were established. A total of 25 tunnels were obtained from the first lateral tunnel near the medial condyle of the proximal femur. Among them, 5 tunnels were placed outside of the femur vault, which did not conform to the actual surgical procedures and were excluded. Therefore, we obtained 20 different femoral tunnel knee models. Among them, tunnel 13 is the PCL located in the middle of the anatomical footprint. The femoral and tibial tunnel diameters were uniformly set at 9 mm (Fig. 3).

**Fig. 4:**
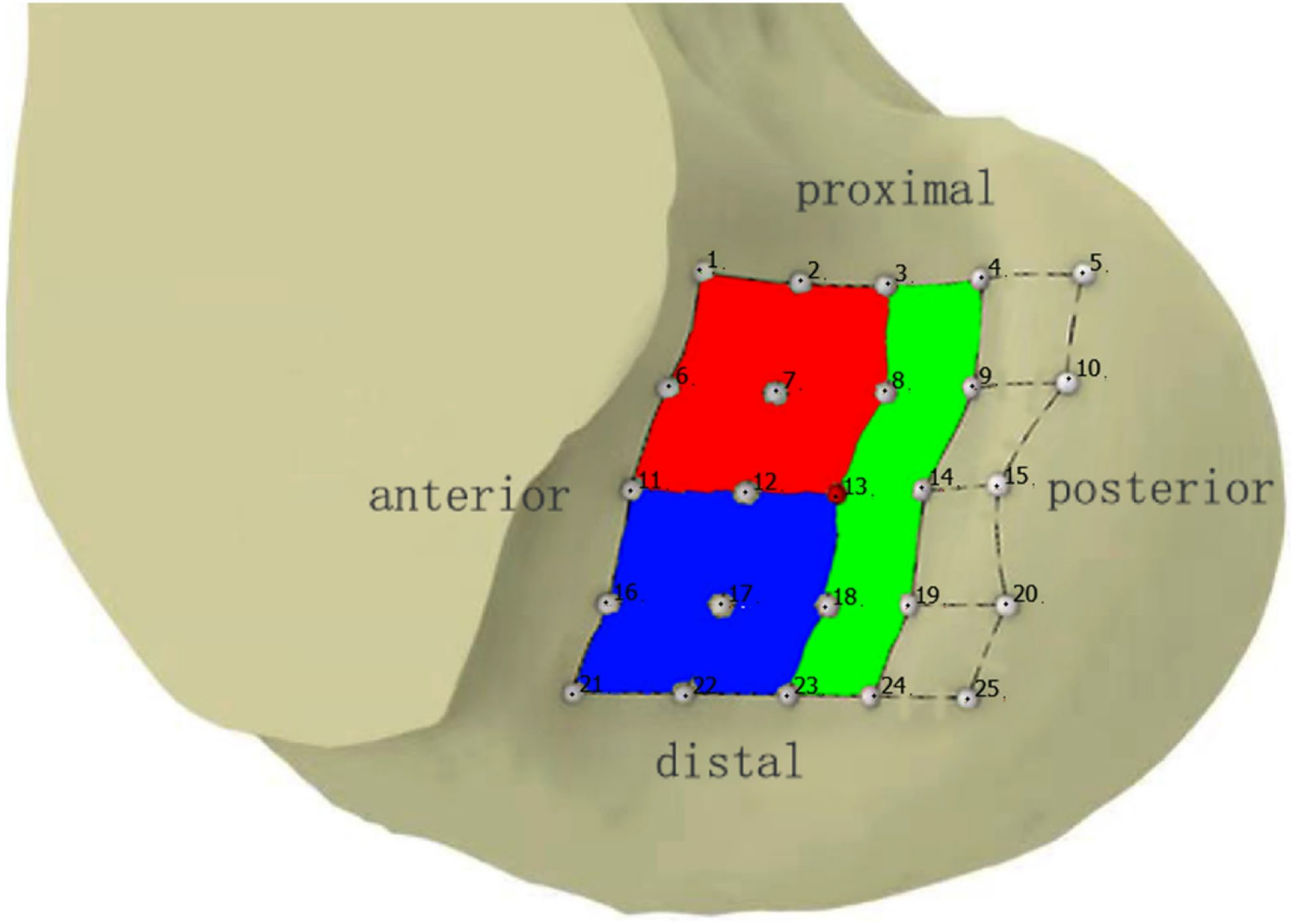
The antero-proximal region is marked red, the antero-distal region is marked blue and the posterior region is marked green

We defined the position of the femoral tunnel relative to tunnel 13. We used the standard anatomical orientation for the partitioning, and we obtained the antero-proximal region, antero-distal region and posterior region (Fig. 4).

### Measurement method

**Fig. 5:**
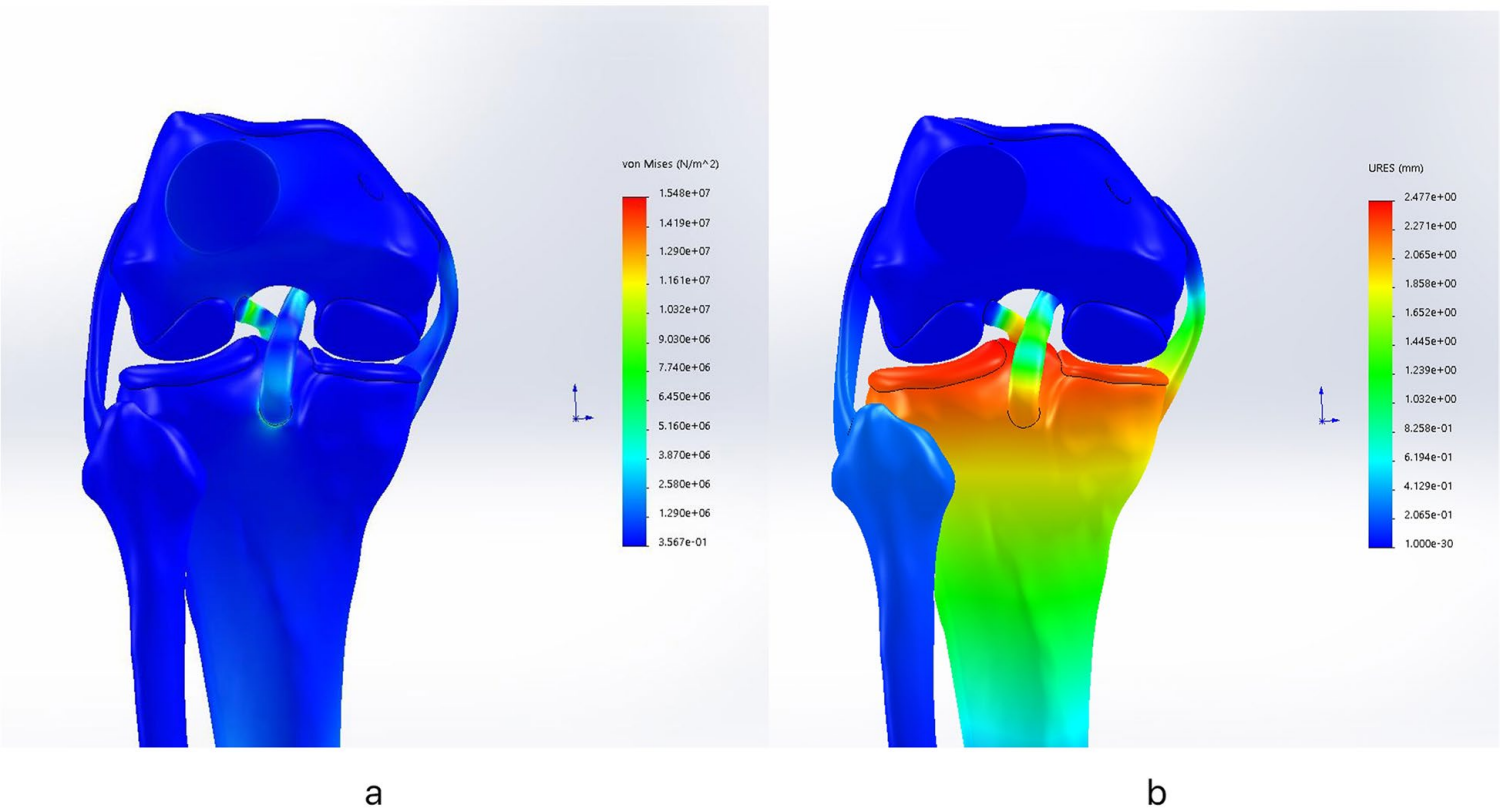
A posterior tibial load of 134 N was applied to the reconstructed model of tunnel 13 with 90° flexion. The peak stress (Fig. 5a) and posterior tibial displacement (Fig. 5b) were recorded

After the PCL reconstruction was complete, a posterior tibial load of 134 N was applied to the reconstructed model, and different tunnels of the femur, peak stress at knee angles of 0°, 30°, 60° and 90° and the displacement of the proximal tibia were recorded and compared. Knee stability was determined by measuring the size of the posterior displacement of the tibia relative to the femur. After the test, the peak stress of the grafts and the posterior displacement values of the tibia in different femoral tunnels were recorded and compared at different knee flexion angles (Fig. 5).

## Results

**Fig. 6:**
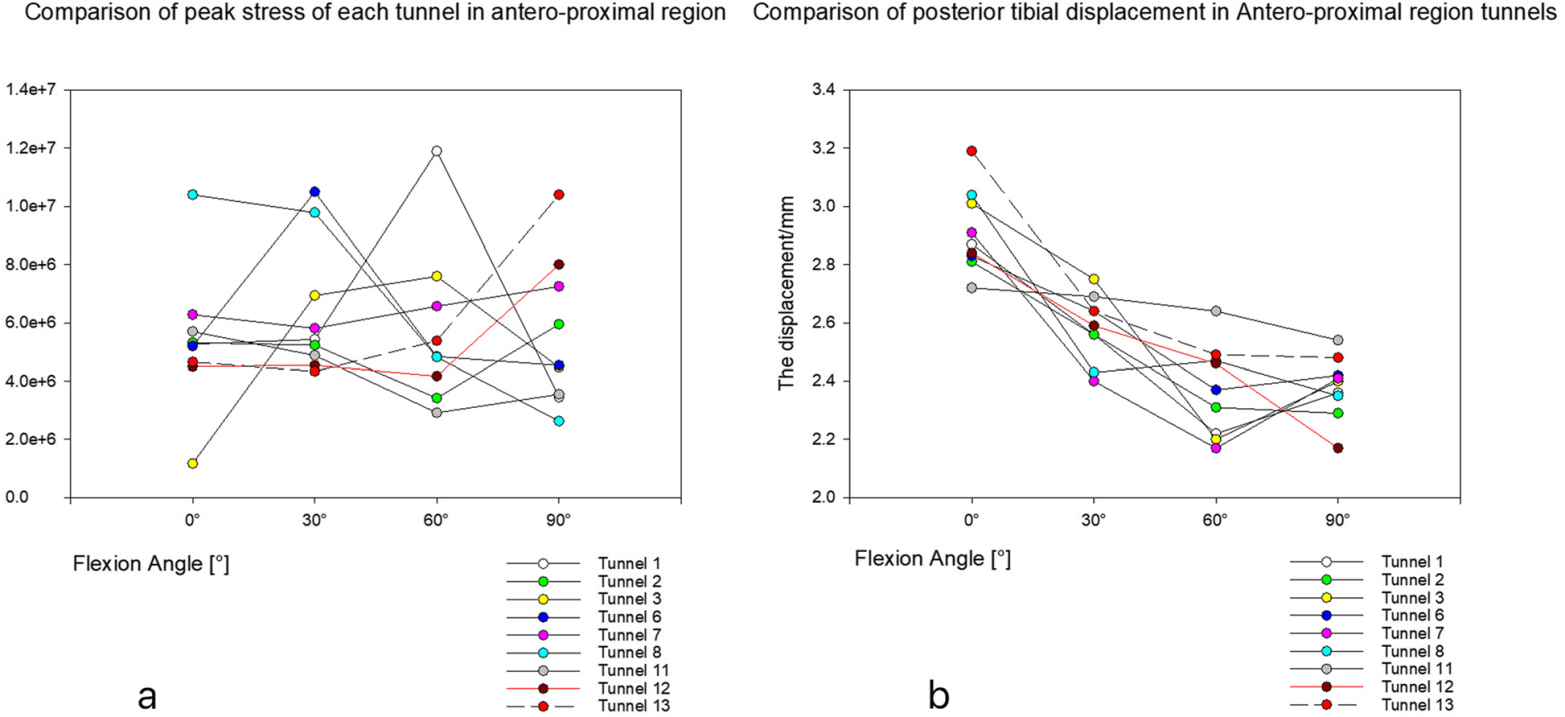
**a** shows the peak stress comparison graph of antero-proximal region tunnels. **b** is a comparison of posterior tibial displacement of tunnels in antero-proximal regions

**Fig. 7:**
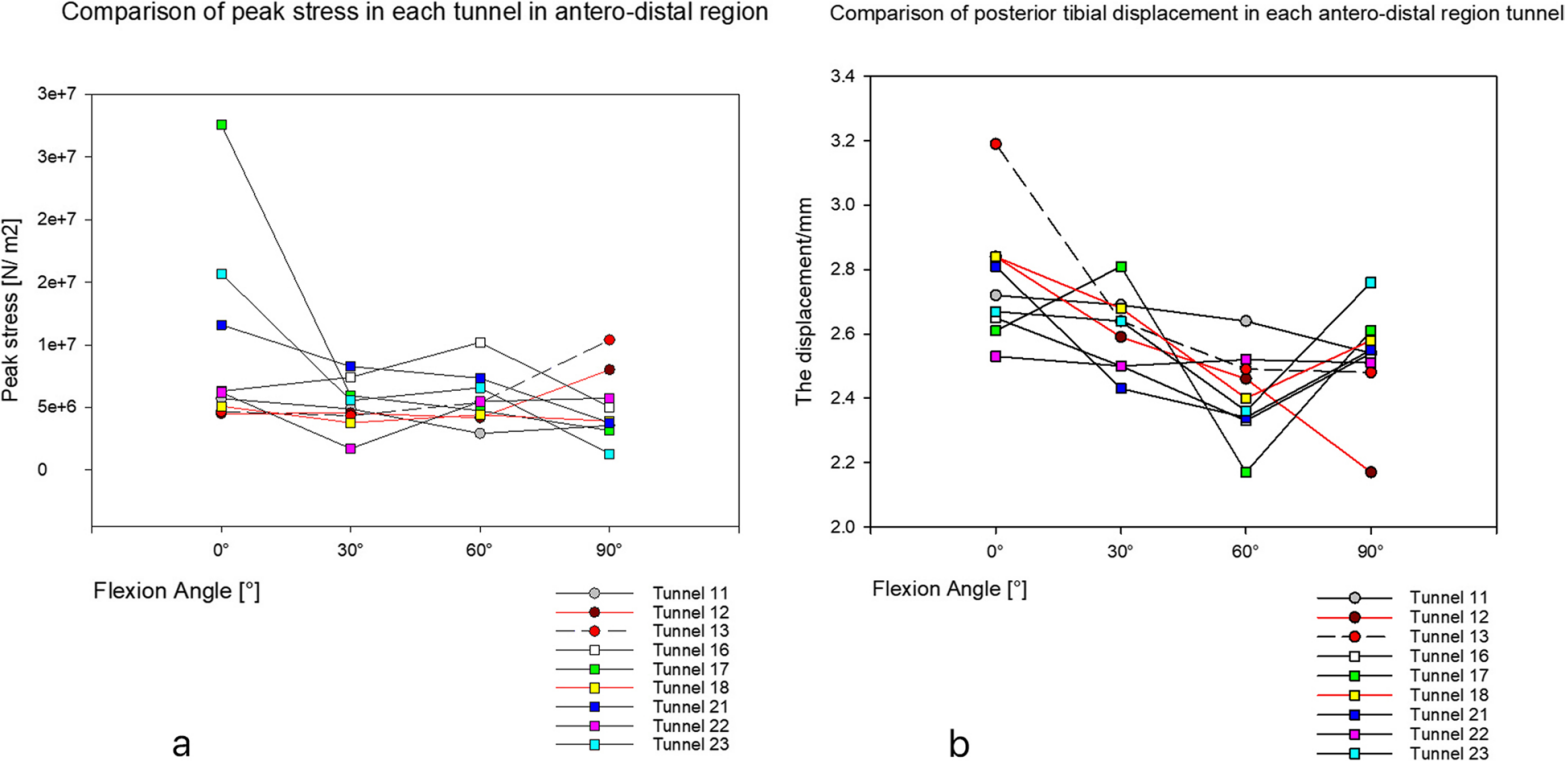
**a** shows the peak stress comparison graph of antero-distal region tunnels. **b** is a comparison of posterior tibial displacement of tunnels in antero-distal regions

**Fig. 8:**
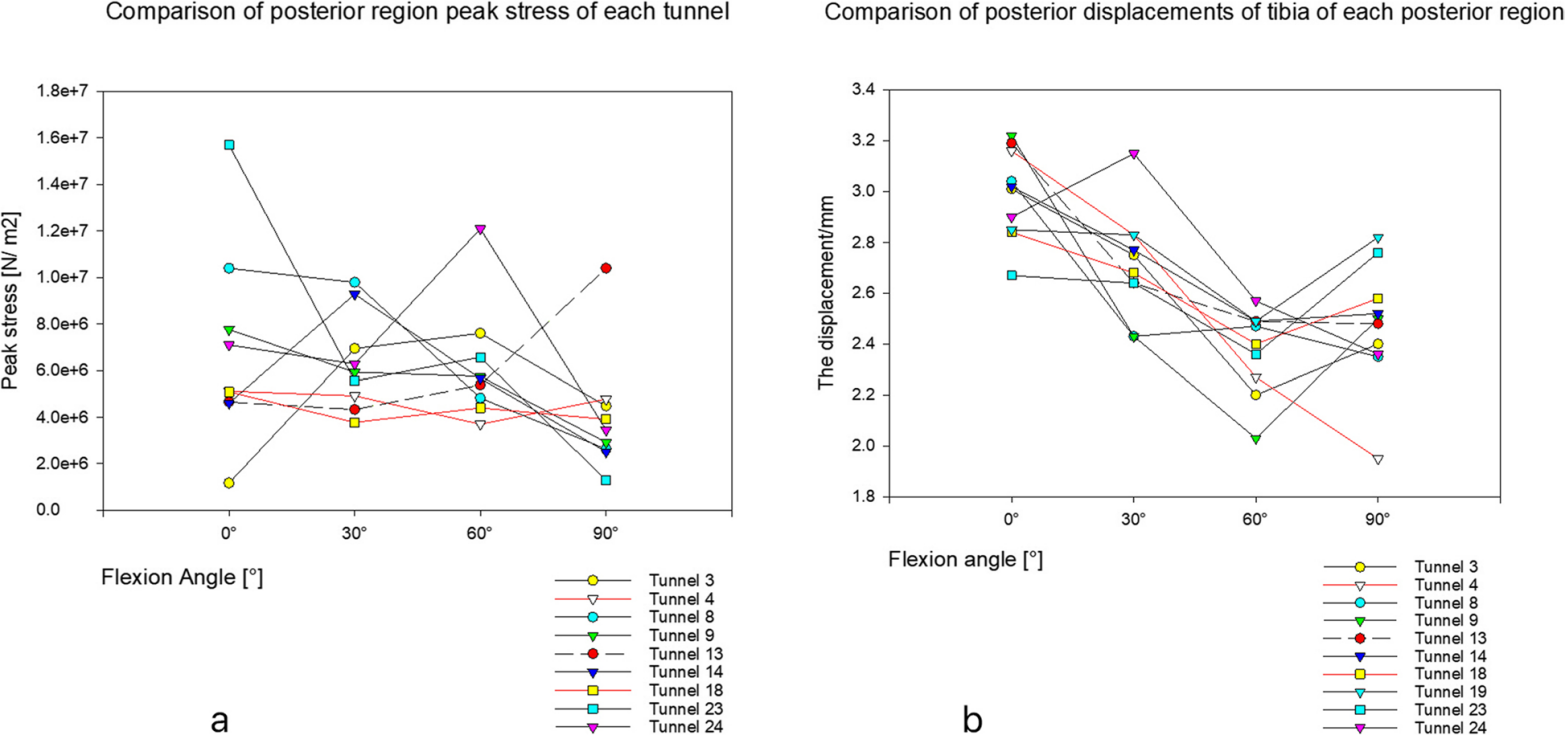
**a** shows the peak stress comparison graph of posterior region tunnels. **b** is a comparison of posterior tibial displacement of tunnels in posterior regions

For the test and comparison of graft peak stress, in the antero-proximal region (in tunnels 2, 6, 8, 11 and 12), the peak stress of the graft was smaller than that of tunnel 13 (but only when the flexion was 60° and 90°). Notably, the peak stress of tunnel 12 was slightly smaller than that of tunnel 13 at 0° and was slightly larger than that of tunnel 13 at 30° (Fig. 6a). In the antero-distal region, the peak stress of grafts in tunnels 11, 12, 17 and 18 was lower than that in tunnels 13 at 60° and 90° of flexion. Furthermore, the peak stress of tunnel 18 was slightly greater than that of tunnel 13 at 0° flexion; additionally, with the increase in bending angle, the difference in peak stress between the two tunnels also increased (Fig. 7a). In the posterior region, the stress peaks of tunnels 4 and 8 were smaller than those of tunnel 13 at 60° and 90° of flexion and were opposite at 0° and 30° of flexion (Fig. 8a).

In the test and comparison of posterior tibial displacement, the displacement value of tunnel 12 was smaller than that of tunnel 13 at different flexion angles (Fig. 6b). The posterior tibial displacement of tunnel 18 was slightly smaller than that of tunnel 13 at 0° and 60° of flexion (but opposite at 30° and 90° of flexion) (Fig. 7b). Moreover, the posterior tibial displacement of tunnel 4 was slightly greater than that of tunnel 13 (but only at 30° flexion), and the other angles were smaller than that of tunnel 13 (Fig. 8b).

Therefore, after a comprehensive comparison, tunnel 12, tunnel 18 and tunnel 4 were selected as the best tunnels in the antero-proximal region, antero-distal region and posterior region, respectively. It is worth mentioning that tunnel 4 is located outside of the intercondylar fossa of the femur, wherein the tunnel is located, which is in contrast to the current surgical approach. Therefore, we ruled this tunnel out. Finally, we only obtained two ideal tunnels (tunnel 12 and tunnel 18).

## Discussion

The present study used a finite element analysis to determine the biomechanical effects of femoral tunnels at different locations on PCL reconstruction. The most important findings of this study are as follows: 1. compared with anatomical footpath tunnel 13, tunnel 12 and tunnel 18 can effectively reduce the peak stress of the graft, especially at high flexion angles (60° and 90°); and 2. compared with anatomical footmark tunnel 13, tunnels 12 and 18 did not increase the posterior displacement of the proximal tibia. Therefore, we hypothesized that tunnel 12 and tunnel 18 could effectively reduce the peak graft stress without reducing the posterior stability of the knee joint.

At present, biomechanical experiments have confirmed that the ‘‘killer turn’’ produced by grafts and tibia tunnels is one of the factors that contribute to the loosening and failure of grafts [35–37]. The “critical corner” or “acute angle”, whereby grafts and femoral tunnels form, is another potential risk factor [38–40]. During PCL reconstruction, an acute graft bending angle is formed at the interarticular interface between the graft and the femoral tunnel. Due to the presence of this acute angle, when the knee joint is stretched and flexed, the graft will constantly rub against the sharp edges of the femoral tunnel hole, thus resulting in excessive wear and stretching of the graft. The proper placement of tunnels to minimize “critical corner” or “acute angle” effects is essential for successful PCL reconstruction [40–42].

In the literature, there are relatively fewer finite element and biomechanical studies on the different positions of femoral tunnels. Seo et al. [27] reconstructed the anterolateral, central and posteromedial tunnels of the femoral footprint by using a single beam of three-dimensional finite element analysis and measured the changes in PCL tension at 0°, 45°, 90° and 135° of the knee bend. From their data, when the knee bends beyond 90°, the central tunnel graft is preferable to the other two types of grafts. The study focused on changes in graft tension and tested more angles of knee flexion than we tested. Since we did not test for knee flexion at 135°, this may be the reason why we cannot draw consistent conclusions. However, other scholars have obtained different conclusions about the biomechanical study of the different femoral tunnels. For example, Markolf et al. [28] conducted a biomechanical study to measure plant tension and length changes at six bend angles in the anterolateral, central and posteromedial regions of the femoral footprint. This study suggested that a posteromedial tunnel should not be used for single-bundle posterior cruciate ligament reconstruction. Moreover, Burns et al. [29] used 7 adult knee specimens to conduct biomechanical research. In the proximal and distal directions of the equidistant point, two femoral tunnels were built at a distance of 5 mm. They concluded that the distal femoral tunnel produced better posterior stability when increasing the knee bend angle than did the proximal or isometric tunnels. This is consistent with our conclusions, and the conclusion of our experiment on Tunnel 18 coincides with the conclusions of this study, except that our trial was designed to test the peak stress of the graft. Similarly, Galloway et al. [43] defined five femoral tunnels in an isometric position (the isometric points were 4 mm apart at the remaining four points) and showed that PCL reconstruction centered on isometric femoral attachment resulted in decreased posterior stability at high knee flexion angles. They concluded that the biomechanical effect of the PCL reconstruction was better when the graft was placed in the distal direction of the isometric point (near the isometric point 1/3 position). In contrast to this study, we did not design the femoral tunnel with a 4 mm spacing, and we used a posterior tibial load of 134 N instead of 100 N. The best location for the femoral tunnel to be established in this study is similar to the tunnel 18 we designed. This result is essentially consistent with our results.

However, there was no study focused on the other areas except for the 5 points. Although the methods and specific steps in our experiments were different, all of the experiments agreed that the best location for PCL for reconstructing the femoral tunnel was in the antero-distal region [28, 29, 43, 44]. Our study identified the specific coordinates of the ideal tunnel in the antero-distal region.

The finite element analysis, which resolves the limitation of traditional measurement tools, is an important method of biomechanical analysis. With the use of a computer design, the femoral tunnel can be extensively built, and small changes can be observed between the tunnels. In this experiment, 20 femoral tunnels were established, which was not limited in number compared with traditional cadaver specimens and which solved the problem of insufficient cadaver specimens. Unlike most current studies, we further considered the influence of the medial and lateral collateral ligaments and the anterior cruciate ligament to make the results more convincing. Moreover, our experiments tested both the peak stress of the graft and the posterior displacement of the proximal tibia.

Our study had the following limitations. First, this analysis only involved the finite element analysis of the anatomical geometry of one subject, and further in vitro biomechanical tests are needed. Second, in this study, only the influence of the femoral tunnel at different sites was explored, and the influence of tibial insertion was not explored. In previous studies, our team explored the influence of different positions of the tibial tunnel on PCL reconstruction [34]. Finally, this was a theoretical analysis that requires further practical clinical verification.

## Conclusions

In summary, among the options for PCL reconstruction of the femoral tunnel, the tunnels located 5 mm distal to the footprint and 5 mm anterior to the footprint could reduce the peak stress of the graft. In addition, they may reduce the "critical corner" and were shown to not reduce the posterior stability of the knee joint.

## Data Availability

The datasets used and/or analyzed during the current study are available. from the corresponding author on reasonable request.

## Abbreviations

3D: Finite element
CT: Computed tomography
FE: Three-dimensional
MRI: Magnetic resonance imaging
PCL: Posterior cruciate ligament
